# Involvement of DAT1 Gene on Internet Addiction: Cross-Correlations of Methylation Levels in 5′-UTR and 3’-UTR Genotypes, Interact with Impulsivity and Attachment-Driven Quality of Relationships

**DOI:** 10.3390/ijerph17217956

**Published:** 2020-10-29

**Authors:** Laura De Nardi, Valentina Carpentieri, Esterina Pascale, Mariangela Pucci, Claudio D’Addario, Luca Cerniglia, Walter Adriani, Silvia Cimino

**Affiliations:** 1 Faculty of Psychology, International Telematic University Uninettuno, I-00186 Rome, Italy; l.denardi@students.uninettunouniversity.net (L.D.N.); l.cerniglia@uninettunouniversity.net (L.C.); 2 Center for Behavioral Sciences and Mental Health, Istituto Superiore di Sanità, I-00161 Rome, Italy; carpentieri.1406747@studenti.uniroma1.it; 3 Department of Medical-Surgical Sciences and Biotechnologies, Sapienza University of Rome, I-00161 Rome, Italy; esterina.pascale@uniroma1.it; 4 Faculty of Bioscience & Technology for Food, for Agriculture and for Environment, University of Teramo, I-64100 Teramo, Italy; pucci.mar80@gmail.com (M.P.); cdaddario@unite.it (C.D.); 5 Department of Dynamic and Clinical Psychology, Sapienza University of Rome, I-00186 Rome, Italy; silvia.cimino@uniroma1.it

**Keywords:** methylation, CpG epigenetic marker, 5’-UTR motifs, internet addiction

## Abstract

Internet influences our communication, social and work interactions, entertainment, and many other aspects of life. Even if the original purpose was to simplify our lives, an excessive and/or maladaptive use of it may have negative consequences. The dopamine transporter (DAT1) gene was studied in relation to addictions, including excessive use of the Internet. The crucial role of DAT1 was previously underlined in modulating emotional aspects, such as affiliative behaviors. The present research follows a new approach based on cross-correlation between (de)methylation levels in couples of CpG loci, as previously shown. We investigated the possible relationships between Internet addiction, impulsivity, quality of attachment, DAT1 genotypes (from the 3′-untranslated region (UTR) variable number of tandem repeats (VNTR) poly-morphism), and the dynamics of methylation within the 5’-UTR of the DAT1 gene. From a normative sample of 79 youths, we extrapolated three subgroups a posteriori, i.e., one “vulnerable” with high Internet Addiction Test (IAT) scores (and high Barrat Impulsivity Scale (BIS) scores; *n* = 9) and two “controls’’ with low BIS scores and 10/10 vs. 9/x genotype (*n* = 12 each). Controls also had a “secure” attachment pattern, while genotypes and attachment styles were undistinguished in the vulnerable subgroup (none showed overt Internet addiction). We found a strongly positive correlation in all groups between CpG2 and CpG3. An unsuspected relationship between the 3’-UTR genotype and a 5’-UTR intra-motif link was revealed by CpG5–CpG6 comparison. The negative correlation between the CpG3–CpG5 positions was quite significant in the control groups (both with genotype 10/10 and with genotype 9/x), whereas a tendency toward positive correlation emerged within the high IAT group. In conclusion, future attention shall be focused on the intra- and inter-motif interactions of methylation on the CpG island at the 5′-UTR of DAT1.

## 1. Introduction

Several recent studies [1,2] showed evidence regarding the different psychopathological risks associated with an excessive use of the Internet. Some of these comorbid symptoms include loss of control over time spent on the Internet, withdrawal symptoms, difficulties in social and emotional relations, and negative mental-health consequences, e.g., other addictions, anxiety, impulsiveness, mood alteration, depression, etc. [3,4]

Nevertheless, Internet addiction (IA) is not yet fully recognized within the spectrum of addictive disorders, although Ivan Goldberg in 1995 and the American Psychiatric Association (APA; [5]) proposed including IA within the Diagnostic and Statistical Manual of Mental Disorder (DSM) in 2013. Moreover, in contrast to other forms of addictive behavior (like gambling and psychoactive substance abuse), only a few studies focused on the genetic predictors and environmental influences of Internet addiction vulnerability.

Consistently with the Developmental Psychopathology framework, insecure attachment was indicated as one of the environmental factors potentially associated with IA, especially in adolescence and young adulthood. In fact, the quality of early experiences is known to influence individual emotion regulation capacity later in life, which is a key aspect affecting the problematic use of the web [6]. Insecurely attached subjects frequently show anxious and/or withdrawn patterns of interactions and interpersonal incompetency. From a general point of view, there are three main subtypes of the insecure attachment style, among which, in particular, “dismissing” subjects feel comfortable without close emotional relationships. Starting from this consideration, it could be questioned whether these subjects would be at risk of developing an IA. Dismissing adolescents and young adults might adopt avoiding strategies to keep distance from their inner affective world, due to the discomfort that both positive and negative profound emotions cause them: they may be eventually seeking for web-mediated interactions, which can be less emotionally laden than face-to-face ones [7,8]. On the other hand, “preoccupied” and “fearful” subjects may appear respectively withdrawn and anxious of being rejected by others [9]. These subjects may attempt to mitigate this preoccupation and fear of missing out by overusing the web, thus seeking external confirmation [6]. 

From a genetic and neurophysiological point of view, attachment styles are linked to changes in the dopaminergic system, as shown by many studies. For example, infant cues, such as smiling or crying facial expressions, are powerful motivators of human attachment behavior, activating dopamine-associated brain reward circuits [10]. Subjects with secure patterns of attachment, compared with insecure/dismissing ones, showed greater activation of regions in the mesocorticolimbic dopaminergic pathway, including the ventral striatum and ventromedial prefrontal cortex, and the oxytocin-associated hypothalamic/pituitary region. In general, securely attached individuals showed also greater activation of the dorsolateral prefrontal cortex, which was previously implicated in cognitive control and habit formation [11]. Results around the activation of different dopaminergic areas of the brain suggested that individual differences in attachment patterns may be linked with development and function of the dopaminergic systems. The emotional and behavioral characteristics associated with the quality of attachment, which models emotion regulation processes, are particularly evident in adolescents; in fact, adolescence is a particularly complex developmental phase generally characterized by hyperactivated mesolimbic dopamine [12], leading to emotional and behavioral deviance, dysfunctional impulsivity, high harm-avoidance, novelty seeking, reward dependence, and low cooperativeness [13,14,15].

A key player in dopamine (DA) neurotransmission is the dopamine transporter (DAT), a protein located at nerve terminals which modulates the dynamics and levels of released dopamine (by recycling extracellular dopamine back into the presynaptic terminal, thus terminating its action). Dysregulated DA activity may stem from altered release or reuptake; therefore, proper regulation of DAT1 gene expression is critical to maintain homeostasis in the dopaminergic systems. The human DAT1 gene, coding for DAT protein, has a polymorphic 40-base pair (bp) variable number of tandem repeats (VNTR) in the 3’-untranslated region (3’-UTR) of the dopamine transporter gene (SLC6A3, on chromosome 5p15.3). Even if the 40-bp VNTR element can be repeated 3–11 times, the greatest frequency polymorphisms are 9- or 10-repeat alleles [16]. Both in vitro and in vivo studies demonstrated that gene expression was greater for the 10-repeat allele than for the 9-repeat allele, showing that this polymorphism is able to affect DA dynamics through protein expression. Moreover, this polymorphism was previously reported to be associated with impulsivity and drug dependence/addiction. Some authors suggested an association between IA and dysfunction of the brain dopaminergic system [17]. Excessive Internet use is associated with an overall reward deficiency related to reduced dopaminergic activity, similar to that present in drug abuse. Cash et al. [18] posited a correlation between the DAT1 gene, in terms of protein functioning and promoter methylation, with the abuse of web resources [18]. Further, studies by Cimino and Cerniglia [6] showed evidence for the crucial role of attachment in modulating dopaminergic aspects, such as the reward and sensation seeking, aggression, and affiliative behaviors.

Recent studies confirmed how the interaction between genetic and environmental factors, and its impact on child and adolescent emotions and behaviors, might be mediated by epigenetic mechanisms of DNA methylation [19]. The methylation of DNA (leading to gene silencing) is one the most studied among epigenetic mechanisms. It is mostly realized within CpG islands, where cytosine conversion to 5-methyl-cytosine shuts down the expressions of genes. Following studies by Tonelli et al. [20], Lambacher et al. [21], and [22], this research exploited our new cross-correlation approach by applying it to the issue of IA disorder. Therefore, considering the prominent role of DA system dysregulation in all addictions, we focused our study on the genetic and epigenetic determinants for derangement of the DAT1 gene, which codes for human DAT protein.

## 2. Method

### 2.1. Subjects

We recruited a community sample of 79 youths composed by 20 males and 59 females, with an age range from 18 to 34 years old and an average age of 23 years old. We looked at the possible role played by dynamics of methylation within DAT1 5’-UTR and DAT1 3′-UTR genotypes, for associations between IA, impulsivity, and quality of attachment. Psychometric data were gathered using the following scales:

- Internet Addiction Test (IAT, Young, 2009 [4]. Cut off references: Young, 1998 [23]).
- Barrat Impulsivity Scale (BIS, Barrat, 1994 [24]. Cut off references: Fossati et al., 2001 [25]).
- Relationship Questionnaire Interview (RQ_I, Bartholomew and Horowitz, 1991 [9]).

DNA samples for genetic studies were gathered from buccal swabs from each subject. Sampling was performed under controlled and standard conditions in a room specifically designed for this purpose within our Department of Sapienza University of Rome, in a time slot from 17:00 to 19:00. From buccal cell samples, biomarker analyses were run with focus (a) on the genotypes originating from a 3′-UTR VNTR polymorphism and (b) on the DNA methylation of loci 1–7 in the 5’-UTR of the DAT1 gene [26]. The research protocol was approved by the Ethical Committee of S.C.’s Department before the beginning of the study (Prot. n. 0000018, dated 09/01/2019, authorized by Sapienza University of Rome). The rules in accordance with the Code of Ethics of the World Medical Association (“Declaration of Helsinki”), which was printed in the British Medical Journal (on 18 July 1964), were fully respected. All subjects signed an informed consent document.

Genomic DNA was prepared from buccal swab samples using the BuccalAmp™DNA Extraction Kit, following the manufacturer’s instructions (Epicentre; Madison, WI, USA). The 3′-UTR repeated sequence of the DAT1 gene was amplified by polymerase chain reaction (PCR), as previously described [26]. The primer sequences employed were 5′-TGT GGT GTA GGG AAC GGC CTG AG-3′ (DAT1-F) and 5′-CTT CCT GGA GGT CAC GGC TCA AGG-3′ (DAT1-R). The PCR amplification was carried out in a final volume of 50 μL, containing 3 μL of genomic DNA (prepared using the DNA extraction kit, see above), 1.5 mM of MgCl_2_, 200 μM of dNTP, 50 mM of KCl, 10 mM of Tris–HCl (pH 8.3), 0.25 μM of each primer, and 1 U of Promega Taq DNA polymerase (Promega; Madison, WI, USA). The PCR amplification was performed for 35 cycles consisting of 94 °C for 45 s, 57 °C for 30 s, and 72 °C for 30 s. The genotype was estimated from the size of the PCR product analyzed by electrophoresis on 6% acrylamide gels stained with ethidium bromide.

**Figure 1:**
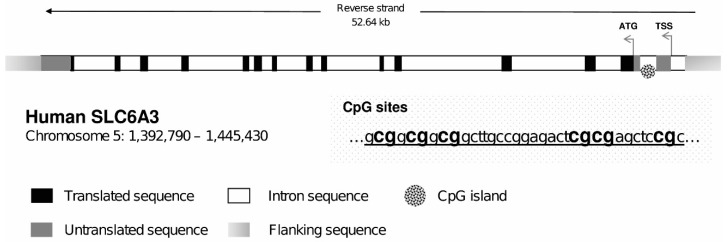
Schematic representation of human dopamine transporter (DAT1) gene. Positions of transcription start site (TSS), translation start code (ATG), and exons and introns are depicted. The CpG island containing the six CpG sites is reported from +712 to +746 relative to TSS, well within the first intron, located in the 5′-untranslated region (UTR) of the gene. The first “cg” of the shown sequence is at position 1,444,717 (at +713), and 1,444,716 (at +714) on the reverse strand of chromosome 5. Taken from Tafani et al. [22].

The level of methylation for each subject was analyzed using PyroMark Q24 Software (Qiagen, Hilden, Germany), which calculates the methylation percentage (mC/(mC + C)) for each CpG site, allowing quantitative comparisons (mC is methylated cytosine and C is unmethylated cytosine). Methylation status at the 5’-UTR of DAT1 (ENST00000270349.12) was determined on bisulfite-converted buccal swab DNA. After extraction, 0.5 μg of DNA from each sample was treated with bisulfite using the EZ DNA Methylation-Gold™ Kit (Zymo Research, Orange, CA, USA) and amplified using the PyroMark PCR Kit (Qiagen, Hilden, Germany), in accordance with the manufacturer’s protocol. PCR conditions were as follows: 95 °C for 15 min, followed by 45 cycles of 94 °C for 30 s, 56 °C for 30 s, 72 °C for 30 s, and, finally, 72 °C for 10 min. The schematic representation of CpG island in DAT1 is illustrated in Figure 1. The 5′-UTR was analyzed using the pyrosequencing assay (PM00022064); all details of the sequence are available on the Qiagen web site (www.Qiagen.com). Two standard human DNA samples of fully methylated (100%) and unmethylated DNA (0%) were purchased from Zymo (Zymo Research; Irvine, CA, USA) and used, respectively, as positive and negative methylation controls. They were bisulfite-converted and were run along with the experimental samples.

### 2.2. Procedure for Subgrouping

First of all, to compose our investigated subgroups, we selected youths that were over a fixed threshold in the IAT (>50) and/or BIS (>67) scales. The first subgroup was composed of 9 people and the second contained 18 people. The first evidence was that the 9 people in the IAT subgroup entirely overlapped those present in the BIS subgroup, in that all people vulnerable to Internet addiction were also suffering elevated impulsivity.

Another important set of data was referred to the attachment patterns referencing the Relationship Questionnaire (RQ_I) (Bartholomew and Horowitz, 1991), a self-report measure in which participants select one of four prototypes containing multi-sentence descriptions of attachment patterns. The prototypes, along with sample sentences, are:

- 1—Secure: ‘‘I feel comfortable depending on others and having others depend on me’’;
- 2—Insecure–dismissing: ‘‘I am comfortable without close emotional relationships’’;
- 3—Insecure–preoccupied: ‘‘I want to be intimate with others, but I often find that others are reluctant to get as close as I would like’’;
- 4—Insecure–fearful: ‘‘I find it difficult to trust others completely, or to depend on them’’.

Youths (*n* = 9) with a score over fixed thresholds (both in IAT and in BIS subgroups) showed attachment patterns quite different from attachment pattern 2 (insecure–dismissing), while people (*n* = 9) with a score over the fixed threshold in BIS but not in IAT showed attachment pattern 2 (insecure–dismissing). 

Attachment pattern 2, interestingly, was shown to act as a protective factor (in impulsive people) against Internet addiction (Table 1). 

Starting from this consideration, we composed a control group for high-IAT people, making some differentiations within the total of 61 people with low-risk BIS scores. Since only two subjects exhibited the 3/3 genotype, we decided not to consider this condition, as it is poorly represented in the general population. With the remaining 59 people, we proceeded by excluding 27 people with attachment pattern 2, because it was nearly completely absent from the high-IAT group. In other words, attachment pattern 2 should not appear in the control group because it was indicated as a protective factor against IA. We aimed to divide the remaining people according to the DAT1 genotype. We considered proper controls to consist only of 24 people who showed pattern 1 (see Table 2). We excluded 8 people who showed attachment patterns 3 and 4, also due to lack of enough cases for all genotype combinations.

The final 24 people were analyzed considering the 10/10 and 9/x genotypes. In this way, three final subgroups were composed, namely, people with high IA score (9 people, of which 3 were 10/10 and 6 were 9/x), control people with low-risk scores, attachment pattern 1, and the 10/10 genotype (12 people), and control people with low-risk scores, attachment pattern 1, and the 9/x genotype (12 people) (Table 3).

### 2.3. Statistical Method

As mentioned above, we analyzed DNA methylation levels in DAT1 5′-UTR. We considered two CpG islands, i.e., motif CGG CGG CGG (M1, M2, M3) and motif CG CG (M5, M6), plus CpG M7. Starting from the methylation data, we built three independent matrixes of cross-correlation for people belonging to the above mentioned groups, through Pearson R value calculation. Within each matrix, pairwise correlations were run. We took pairs of loci (even non-consecutive across motif, both methylated), with each position compared with all other positions [22]. A significant (*p* < 0.05) correlation was only considered for an absolute R value of >0.5529, while a significant tendency (0.10 < *p* < 0.05) was also considered for an absolute R value of >0.4762 (these pairwise correlations were considered «present» with acceptable risk of a false positive, as reported in Table 3). 

Correlations below these thresholds, however, are not necessarily absent, as the chance of finding false negatives was high. Hence, pairwise correlations were considered «absent» with acceptable risk of a false negative for an absolute R value of <0.3646 (these correlations are reported in Table 3). A number of all these pairwise correlations for 0.3646 < R < 0.4762 were classified as «undetermined» (see $ in Table 3), since there was too high a probability to commit either a false positive or a false negative. 

Once all pairwise correlations were classified as “present”, “absent”, or “undetermined” independently in each matrix, we compared the matrixes to see what correlations were “present” in a phenotypic subgroup and “absent” in another phenotypic subgroup. Starting from the synoptical comparison of the three matrixes, we composed Table 3 to show the significant differences between the subgroups for certain correlations. We limited our analysis to the “most important” CpGs. In this way, we aimed to investigate which setup was the most probable in each subgroup when considering CpG dynamics. A positive correlation between loci means that as far as one locus increased, the other one also increased, whereas negative correlations represent an anti-correlation, i.e., when one locus increased, the other one decreased. In the case of an absence of correlation, each locus of the pair was considered to be independent from the other.

## 3. Results

Based on the Pearson R values calculated for each subgroup on raw methylation scores, we compared information about all correlations across subgroups (Table 3). The only pairwise correlation found to be strongly positive in all subgroups was between M2 and M3 (R values of 0.901 for high IAT, 0.832 for 10/10 controls, and 0.771 for 9/x controls): being positive in all subgroups, therefore, it was not useful for a discrimination between subgroups. This confirmation of previous results (see Carpentieri et al., submitted) showed that methylations at loci 2 and 3 are interrelated. 

M5–M6 was a positive pairwise correlation shown in high IAT subjects and controls with genotype 9/x, but no evidence for this intra-motif methylation was found in controls with genotype 10/10. This is the first report of an association between the 3’-UTR genotype and a 5’-UTR intra-motif link. 

M3–M5 pairwise correlation was found to be negative in both control subgroups but absent in the IAT group. Furthermore, while an anti-correlation with a negative R value was clear in controls, a tendency toward positive correlation emerged within the high IAT group.

**Figure 2:**
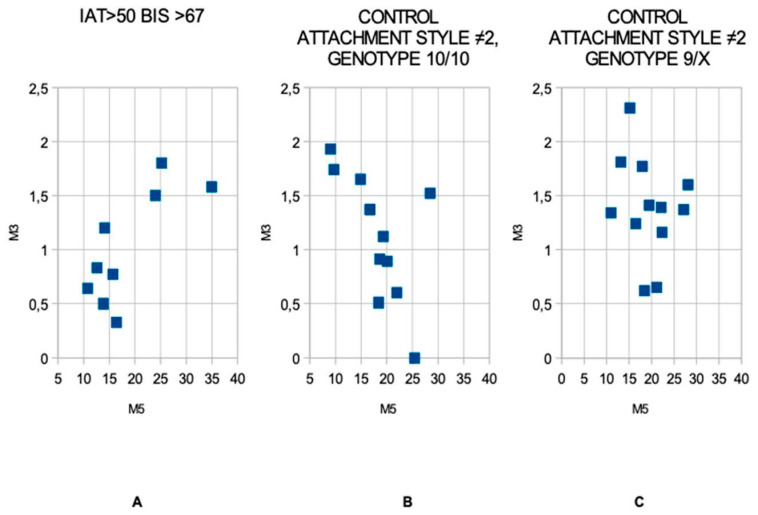
Cross-correlation between CpG3 and CpG5: the high IAT (IAT > 50 and BIS > 67) group (panel **A**), seems to show a positive correlation, while control groups matched to attachment style 2, both with genotype 10/10 (panel **B**) and genotype 9/x (panel **C**), show negative correlations. Note that the mean methylation levels, expressed in % of methylated cytosine over total cytosines, are similar in all subgroups. In graphic for genotype 10\10 (panel **B**) only 11 points were marked instead of 12 due to two subjects having the same M3 and M5 values and, therefore, occupying the same position.

The following scatterplot graphics (Figure 2) show the results discussed above.

## 4. Discussion

Several studies underlined that saliva samples with higher epithelial content were more similar to brain with regard to DNA methylation levels [27,28]. Significant and concordant changes in DNA methylation were found for several genes in both saliva and brain, making saliva a valid source for methylation studies in several diseases [29,30].

Traditional studies in epigenetic research underline the importance of the complex interaction between the DAT1 gene and the environment [31]. Our innovative approach refers to the study on DAT1 5’-UTR based on cross-correlations, showing that specific patterns exist in the dynamics of CpG methylation. The results of our recent studies showed that parkinsonian patients were characterized by an overall hypermethylated condition in the 5’-UTR [22,32], opposite from what was found in ADHD adolescents, who showed hypomethylated conditions [20,21].

Cross-correlations shown by Tafani et al. [22] considered evidence of both a methylated and a de-methylated loci status. Among the different matched loci, significant relationships showed specific dynamics among the CpGs within and between the two motifs.

Our approach investigated simple pairwise correlations between couples of individual CpGs methylated at the same time. Our focus was on subjects with high IAT scores out of a normative sample recruited among adolescents and young adults. At this age, hyperactivation derives from neurobiological plasticity, which allows adolescents to adjust to the several body and emotional transformations they undergo, including physical changes, emotional experiences, separation from internalized parental figures, and construction of a personal, social, and sexual identity [33,34,35]. Therefore, youths in this phase could be at higher risk for psychopathology (including depression, anxiety, problematic use of the web, and Internet addiction).

Starting from the whole sample, a subgroup of high IAT subjects was compared with two control groups, IAT < 50 and BIS < 67, the first with genotype 10/10 and the second with genotype 9/x: moreover, both were characterized by a secure relationship attachment style, formalized as a control situation by definition; this attachment pattern was also found in some high IAT subjects. Only one attachment style, the insecure–dismissing, was not found at all in the high IAT group, but only in matched subjects over the BIS threshold. Therefore, this style was considered to act as a protective factor against IA disorder.

A core relationship was found between CpG2 and CpG3 in the high IAT group as well as both control groups, meaning that as far as locus 3 was methylated, locus 2 was also. An identical situation occurred between CpG2 and CpG3 in a normative study by Carpentieri et al. (submitted [36]), demonstrating a significant intra-motif link. The intra-motif relationship between positions 2 and 3 was also found in nearly half of the subjects studied by Tafani et al. [22], specifically in those individuals with low levels of methylation at CpG6. CpG5 also appeared to be significantly matched with CpG6 in controls with genotype 9/x, differently from controls with genotype 10/10, for which a matched methylation was absent. This suggested a different involvement of genotype at the 3’-UTR of the gene, revealing an unsuspected relationship with 5′-UTR.

CpG3 plays a particular role together with CpG5, with evidence of anti-correlation in all control groups showing that as far as one locus increased, the other one decreased and vice versa. This result was found when analyzing the externalizing subgroup of a normal population by Carpentieri et al. (submitted [36]), where CpGs1, 2, and 3 assumed the opposite tendency of CpG6, with the first motif opposite to the second one. Parkinsonian subjects developed anomalous links, e.g., CpG2 with 6 and CpG3 with 5 [22]. The negative correlation between motifs (position 3 matched with 2 and 5 matched with 6) was significant in the current control group with genotype 10/10 and quite significant in the genotype 9/x (both control groups). 

Quite differently in the high IAT group, such links in methylation levels were absent. In Carpentieri et al. (submitted [36]), a positive match was found between CpG3 and 5 in the externalizing subgroup. The relationship between externalizing behaviors and Internet addiction was highlighted by different authors. For example, Ko et al. [37] demonstrated how some aspects of IA, such as online chatting, adult sex web viewing, online gaming, online gambling, and the Bulletin Board System are all associated with aggressive behaviors. The aspects of Internet addiction that lead to aggressive behaviors are externalizing phenotypes included in Achenbach’s definition [38]. Therefore, we can hypothesize that the same positive relationship between CpG3 and CpG5 can be found in both the externalizing subgroup of the previous work [36] and in the high IAT subgroup of the current work. From scatterplot graphics, methylation levels in positions 3 and 5 seem to follow an ascendent line in the high IAT group and are negatively matched in control groups, particularly in genotype 10/10.

Different values were also observed in the cross-correlation of positions 1 and 7, i.e., while in the high IAT group no correlation was observed, one was significantly present in the control group with genotype 9/x. This correlation was also found to be positive in a recent study by Tafani et al. [22]. This cross-correlation was not taken into further consideration because of the physical distance between the two loci.

## 5. Conclusions

In contrast with research on parkinsonian patients followed by Tafani et al. [22] and on healthy people considered by Carpentieri et al. (submitted [36]), in this work we investigated simple pairwise correlation between couples of CpGs in IA. The new approach based on cross-correlation of CpG loci, which was already studied on ADHD [21] and Parkinson’s disease [22], was applied in this research focused on vulnerability to Internet addiction disorder. Some of these results found confirmation from other previous research, for example, the positive correlation between CpG2 and CpG3 which was also found in studies by Carpentieri et al. [36] and by Tafani et al. [22]. For the first time, an association was noted between the 3’-UTR genotype and a 5’-UTR intra-motif link (from the CpG5–CpG6 comparison).

This work, together with our previous research, shows that specific patterns exist in the dynamics of methylation. These dynamics are subject to different factors, such as impulsivity, genotype, and relationship attachment.

It is important to underline, however, that IA is a relatively novel clinical manifestation and this research field should consider results from new, more particular studies based on more specific types of web use. There are not consolidated data yet, and current definitions [39] do not constitute a theoretical framework of which to make reference [40]. The label itself of IA is still in question since there is not yet a general definition but many different ones, like compulsive internet use, problematic use of the web, pathological internet use, etc., which are all currently in use in the scientific literature [41,42].

There are only two instruments which were properly built to measure IA, specifically, the Internet Addiction Test [4,43,44], which utilizes dependency criteria to formulate a diagnosis, and the Chen Internet Addiction Scale [45], realized with the aim to screen this phenomenon in adolescents. Several authors suggested the need to find a new, feasible tool for the measure of IA, especially in adolescence [40]. For this reason, the present study was limited by general information on IA vulnerability, without specific data regarding clinical cases.

## Figures and Tables

**Table 1:** Contingency matrix for people in the sample trespassing the impulsivity threshold.

Attachment Style	BIS > 67 IAT > 50	BIS > 67 IAT < 50	Total
1. Secure	3	1	4
2. Dismissing	3	8	9
3. Preoccupied	3	0	3
4. Fearful	2	0	2
Total	9	9	18

BIS: Barrat Impulsivity Scale; IAT: Internet Addiction Test.

**Table 2:** Contingency matrix for people in the sample below the impulsivity threshold.

DAT Genotype	Style				Total
	1. Secure	2. Dismissing	3. Preoccupied	4. Fearful	
10/10	12	15	-	2	29
9/10	9	9	3	-	21
9/9	3	3	1	2	9
Total	24	27	4	4	59

DAT: dopamine transporter genotype could bear 10-repeat or 9-repeat alleles at the 3′-UTR (VNTR polymorphism).

**Table 3:** Presence of positive or negative correlation.

Subgroup	High IAT Score (BIS > 67; IAT > 50)	Control, Attachment Style 1	
DAT Genotype	10/10 (*n* = 3); 9/x (*n* = 6)	10/10	9/x
M5/M6	+0.774 (*)	0.045	+0.676 (*)
M1/M7	0.11	0.424 ($)	+0.596 (*)
M3/M5	0.32	−0.57 (*)	−0.51 (*)

“Presence” of clear correlations in methylation between loci was evidenced by an absolute R value of >0.4762 (see *), while “absence” of clear correlation was evidenced by an absolute R value of <0.3646; if neither present nor absent, “undetermined” was attributed for R values inbetween these thresholds (see $).
